# The complementary roles of conformity and Q-learning in the prisoner's dilemma with punishment

**DOI:** 10.1098/rsif.2026.0124

**Published:** 2026-10-21

**Authors:** Litong Fan, Li Liu, Li’li Liu, Hao Xu, Shupeng Gao, Zhen Wang

**Affiliations:** ^1^ College of Information Engineering, Northwest A&F University, Yangling, Shaanxi, People's Republic of China; ^2^ School of Mechanical Engineering, Northwestern Polytechnical University, Xi'an, People's Republic of China; ^3^ School of Cybersecurity, Northwestern Polytechnical University, Xi'an, People's Republic of China; ^4^ Academy of Mathematics and Systems Science, Chinese Academy of Sciences, Beijing, People's Republic of China; ^5^ Tangshan Research Institute, Beijing Institute of Technology, Tangshan, Hebei, People's Republic of China

**Keywords:** punishment, prisoner's dilemma game, complementary, conformity, *Q*-learning

## Abstract

Punishment has been demonstrated to be an effective mechanism for promoting cooperation, both theoretically and experimentally. However, most previous studies have focused on a single update rule, which fails to reflect the diversity of real societies arising from cognitive heterogeneity. Therefore, this paper investigates the evolutionary dynamics of the prisoner's dilemma with punishment by incorporating Q-learning and conformity update rules. These two rules capture two typical types of individuals commonly observed in real societies: profit-seeking explorers and conservative conformists. We show that an appropriate mixture of the two update rules promotes cooperation through complementary roles: conformity forms clusters to protect cooperation, while Q-learning drives cooperators’ expansion with the help of punishment. This complementarity weakens when conformists dominate. Moreover, we demonstrate that our results are robust across different network structures and punishment parameters. Our findings demonstrate that an appropriate mixture of Q-learning and conformity update rules maximizes the effectiveness of punishment in promoting cooperation.

## 1. Introduction

Cooperation is a crucial factor in the progress of nature and human society, as both groups and individuals benefit from cooperation to achieve success [1–3]. However, fostering the emergence of cooperative behaviour among egoists remains a significant challenge. Since cooperation incurs a cost c to give a benefit b to the opponent while defection incurs nothing, such altruistic behaviour puts individuals at a disadvantage under natural selection [4,5]. To investigate the emergence and maintenance of cooperation among selfish individuals, evolutionary game theory provides extensive mathematical models and analytical tools to uncover the mechanisms underlying the evolution of cooperation [6–8]. Building on this theoretical foundation, researchers have explored the evolution of cooperation from multiple perspectives, ranging from game models and network structures to strategy update rules and underlying mechanisms [9–12]. Over the past decades, researchers have introduced a third type of strategy that provides additional options for agents, allowing the evolution of cooperation to be studied from a more realistic perspective [13–15]. These strategies include reward [16,17], punishment [18,19], voluntary participation [20–22], exit strategy [23], extortion strategy [24] and induction strategy [25]. Among these, punishment (Pu) is an effective mechanism in which cooperators can incur a cost to impose a fine on defectors for their free-rider behaviour [26]. Previous studies have shown that punishment can maintain cooperative relationships or establish cooperative norms [27,28]. Punishment can generally be classified into peer punishment and pool punishment [29]. Here, we focus on peer punishment. Previous studies on peer punishment, both theoretical and experimental, have focused mainly on public goods games, while those on the prisoner's dilemma are relatively rare. Wang *et al.* [30] incorporated punishment as a third strategy in the two-person evolutionary games to investigate its impact on the evolution of cooperative behaviour. Yang *et al.* [31] proposed a symmetric punishment mechanism in which an individual will punish neighbours who hold opposing opinions to foster cooperation. In addition, several studies have proposed various punishment methods to promote cooperation by imposing a direct fine on defectors [32] or by introducing a penalty coefficient to strategies whose pay-off is lower than the neighbourhood average [33]. The availability of punishment makes individuals more inclined to cooperate, as they are aware that their opponent may resort to costly punishment [34].

Many studies on punishment in evolutionary game theory are based on pairwise pay-off comparison updating rules [33,35]. In such rules, individuals update their strategies by imitating those with higher pay-offs. Imitation is a standard approach used to capture evolutionary dynamics in microorganisms, supported by experimental studies on bacterial and viral interactions [36,37]. However, for humans and other social animals, individuals often exhibit conformity, that is, they tend to adopt the strategy chosen by the majority, ensuring that their pay-off will not be much lower than average [38,39]. Several researchers have suggested that a certain proportion of conformists can facilitate the maintenance of cooperative behaviour [40–42] and also facilitate the compliance of social norms [43,44]. Subsequently, other mechanisms were introduced on this basis to investigate their combinatorial effects with the conformity strategy [45,46]. However, research on punishment under conformity rules is rare [47]. In addition, there are also some humans and social animals who exhibit independent thinking abilities and insights in their decision-making processes [48,49]. Reinforcement learning algorithms are very suitable as tools for simulating the strategy update of intelligent agents [50,51], in which agents iteratively update their strategies based on experience to maximize their expected rewards [17]. Researchers investigate the impact of reinforcement learning on cooperation from theoretical or simulation-based perspectives [52,53]. Subsequently, various mechanisms are introduced to examine their effects on the evolution of cooperation under reinforcement learning algorithms, thereby helping to deepen our understanding of the evolution of cooperative behaviour [54,55]. Zhao *et al.* [32] explored the punishment under reinforcement learning and revealed the continuous and discontinuous phase transitions dependencies on punishment strength, cost and game parameters. Zhang *et al.* [56] proposed a layered strategy updating mechanism that combines the Fermi rule with Q-learning to investigate the impact of punishment on cooperation, revealing complex dependencies of cooperation on punishment cost, fine and discount factor parameters.

Therefore, using these two update rules to mimic two typical individuals in society is reasonable and necessary, which explores the evolution of individual behaviour to explain the emergence of cooperation in a more realistic framework. Studying the evolution of cooperative behaviour under the interaction of two update rules is a classic approach in evolutionary games [57]. Previous research has integrated mixed rules with both pay-off-driven and conformity-driven strategy update procedures, demonstrating that the coexistence of multiple update rules facilitates the expansion of cooperation within populations [58,59]. As reinforcement learning has progressively advanced, studies have also examined the combination of reinforcement learning with pay-off-driven update strategies, revealing that an appropriate proportion of reinforcement learning algorithms can enhance cooperation rates [60]. Nevertheless, the combination of reinforcement learning with conformity-driven updates remains an area without a definitive conclusion. In addition, the effect of punishment under these two updating mechanisms is still unknown.

Here, this paper considers the spatial prisoner's dilemma with punishment in which conformity and Q-learning update rules coexist in the population. The Q-learning rule models agents with self-reflective capabilities that adjust their strategies based on past experiences. Conversely, the conformity update rule represents non-reflective agents who update their strategies by following the most common strategy in their neighbourhood. These two types of agents are evenly distributed across the spatial network and correspond to two widely observed categories of individuals in society: profit-seeking actors and conformity-driven conservatives. We investigate the impact of punishment on cooperative behaviour under the condition of different proportions of two types of agents. Our findings indicate that a moderate proportion of their mixture can effectively enhance the promoting effect of punishment on cooperative behaviour. Conformists can form clusters to protect cooperators, while Q-learning agents can utilize punishment to expand cooperator clusters. The complementary effect is strengthened at moderate mixtures and weakened when the density of conformists becomes excessively high. Conformity captures human-like social behaviour, whereas Q-learning represents machine-like, interest-driven decision-making. Our method sheds light on how to model human–machine hybrid prisoner's dilemma games.

The remainder of this paper is organized as follows. Section 2 presents the foundational model, including the game model and the two strategy update rules. Section 3 presents the numerical simulation results. We offer conclusions in §4.

## 2. Model

In the context of playing the prisoner's dilemma game with punishment on a square lattice with periodic boundary conditions, each agent has three options: cooperation (C), which generates benefits for others; defection (D), which maximizes self-interest; punishment (Pu), which sanctions free-riders at a personal cost. Punishment is a special form of cooperation. Punishers behave like cooperators when interacting with other cooperators or punishers but punish defectors by incurring a cost to impose a fine, where the fine is larger than the cost. For simplicity, we employ the weak prisoner's dilemma game, in which the pay-off parameters satisfy T≥R>P=S, with T=b(1≤b≤2), R=1 and P=S=0. The pay-off matrix of the weak prisoner's dilemma game with punishment is listed in table 1.

**Table 1.** Pay-off matrix of the weak prisoner's dilemma game with punishment.

	C	D	Pu
C	1	0	1
D	b	0	b−β
Pu	1	−η	1

In table 1, η is the cost of punishment and β is the fine for a defector. The punishment parameters must satisfy β≥η. The pay-off of agent i is the cumulative pay-offs of all interactions with its neighbours, which can be represented as

(2.1) $$p_{i}=\sum_{j\in N_{i}}p_{ij},$$

where $p_{ij}$ is the pay-off that agent i interacts with agent j and $N_{i}$ denotes the agent i's neighbours set.

The agents are composed of two types of update rules: Q-learning agents and conformity agents. They are uniformly distributed across the network, with their proportion determined by a parameter θ. The proportion of conformity agents is θ, while the proportion of Q-learning agents is 1−θ.

For Q-learning agents, they make decisions based on rewards obtained through repeated interactions with their neighbours and store the state-action corresponding values in the Q-value, which constitutes the Q-table. Subsequently, the agent chooses the action according to the Q-table. For each agent, both its state space and action space are defined as s=a∈{C,D,Pu}, making the Q-table a matrix of 3×3. Although their elements are the same, the state represents the current strategy of the agent, while the action denotes the agent's future choice. All Q-values are initialized to zero, which reflects the assumption that the environment is initially unknown to all agents. The following formula is the updating rules of Q-values:

(2.2) $$Q_{i}(s,a)=Q_{i}(s,a)+\alpha [r_{i}+\gamma \max_{a^{\prime }}Q_{i}(s^{\prime },a^{\prime })-Q_{i}(s,a)],$$

where $Q_{i}(s,a)$ denotes the state-action value for agent i, representing the expected return of taking action a in state s. In each state, the agent chooses the action that has the highest Q-value. Here, α∈(0,1] denotes the learning rate that decides the speed of Q-value towards new information, $r_{i}$ is the reward (pay-off in this game), γ∈[0,1) is the discount factor, which determines the importance of future reward in Q-value update and $\max_{a^{\prime }}Q_{i}(s^{\prime },a^{\prime })$ is the maximum Q-value in next state $s^{\prime }$.

The agent adopts ε-greedy strategy for action selection. Specifically, with probability ε, the agent selects a random action; otherwise, it chooses the action with the maximal Q-value. The value of ε is constant. We set ε = 0.02 in this context.

Conformity agents tend to choose the strategy most prevalent within their local interaction range. Consequently, agent i updates its strategy using the following equation:

(2.3) $$W(N_{s_{i}}-k_{h})=\frac{1}{1+\exp\left((N_{s_{i}}-k_{h})/K\right)},$$

where $N_{s_{i}}$ is the number of neighbours adopting strategy $s_{i}$ of the agent i’, while $k_{h}$ is the half degree of the player i. Here, K=0.1 denotes the selection intensity, which quantifies the irrational choices in the decision-making process. If the number of cooperators among its neighbours equals the defectors’, a conformity agent changes its strategy with probability 1/2.

All procedures are implemented using Monte Carlo simulations (MCs). The stationary fraction of strategies is obtained by averaging the last 5000 MCs of a total of 50 000 MCs. In addition, the final results are averaged over 10 independent runs to ensure sufficient accuracy.

## 3. Results

First, we explore the cooperative behaviour of those two mixed agents with different update rules. In this framework, punishment is regarded as a special form of cooperative behaviour. Figure 1 illustrates the overall cooperation level affected by the density of conformity agents θ and the temptation to defect b. Although pure Q-learning agents perform well in maintaining cooperation, the addition of conformity agents significantly increases the cooperation level. Cooperation peaks when conformity agents constitute approximately 40% of the population. When the density of conformity agents exceeds this threshold, the cooperation level decreases, eventually reaching its lowest point at 80% conformity agents (figure 1a). Moderate introduction of conformists promotes cooperation, but excessive conformist density undermines the cooperation rate. This effect remains strong in the Q-learning subpopulation because the cooperation level within this subpopulation declines more slowly than the proportion of Q-learning agents (figure 1b). In the subpopulations of conformity agents, individuals become unbiased, with cooperation, defection and punishment occurring with equal probability, so the cooperation level eventually reaches 0.6 (figure 1c).

**Figure 1.**
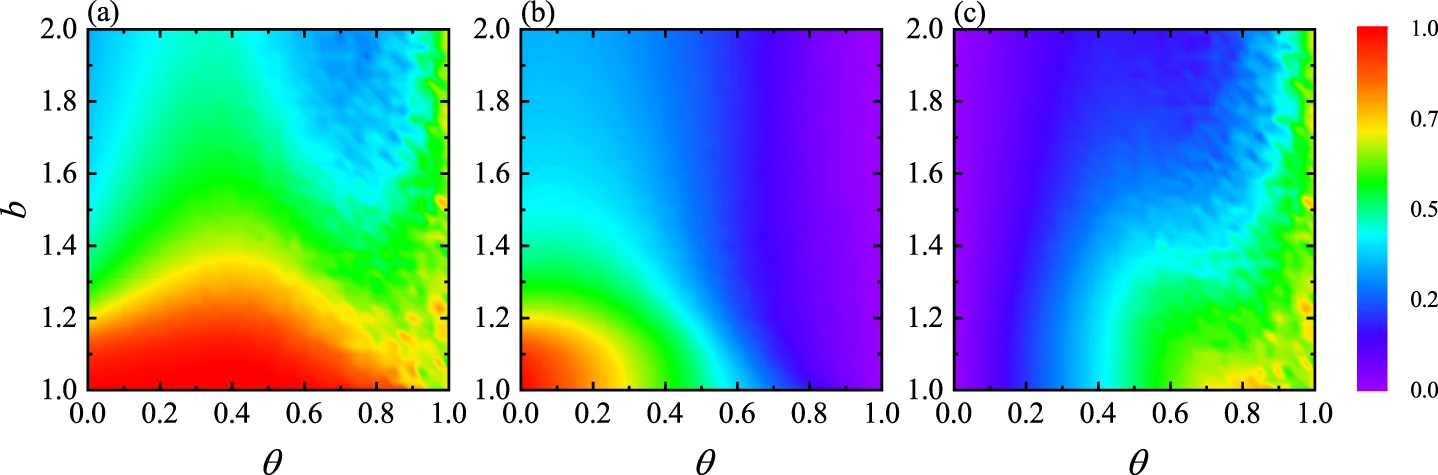
Fraction of cooperators and punishers reaches a peak under θ=0.4 and a trough under θ=0.8 in mixed communities. From panels (a) to (c), the stationary fractions of cooperators and punishers are shown as functions of temptation b and the density of conformity agents for the mixed population, the subpopulation of Q-learning agents and the subpopulation of conformity agents. All results are obtained for β=0.5,η=0.1,α=0.8 and γ=0.8.

To explore why different proportions of conformists influence the cooperation level, we further analyse the evolution of three types of strategy with respect to the temptation to defect b under three different values of θ (0, 0.4, 0.8). It can be seen that when θ= 0.4, the cooperation level decreases most slowly as b increases. The point where the fraction of cooperators intersects with the fraction of defectors is larger than those two (figure 2a,c), delaying the dominance of defectors until b>1.5. In addition, with an increasing proportion of conformists, the cooperation level oscillates with b, because conformity agents are insensitive to pay-offs. Although the coexistence point between cooperators and defectors occurs at a higher value than the case with purely Q-learning agents, the fraction of punishers is notably reduced, leading to a decrease in the overall cooperation level compared with the purely Q-learning scenario when b is small.

**Figure 2.**
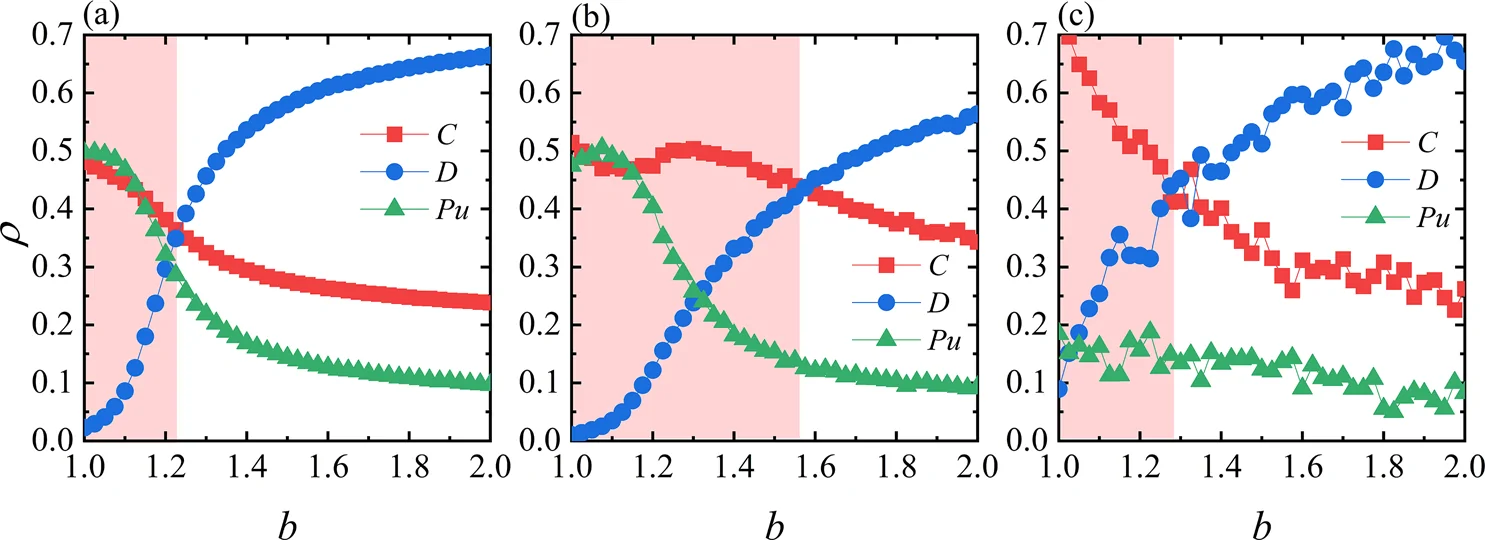
An appropriate proportion of conformity agents increases the threshold for alternating dominance between cooperators and defectors, allowing cooperators to dominate the population even under the high temptation to defect b. (a) In the population of pure Q-learning agents, θ=0, the fraction of cooperators and defectors intersects at around b=1.2. (b) When there is a low density of conformity agents, θ=0.4, the intersection point between the fraction of cooperators and defectors is delayed to around b=1.6, with cooperators predominating before this point. (c) When conformists become the majority, θ=0.8, the intersection point between the fraction of cooperators and defectors occurs at around b=1.3. All results are obtained for β=0.5,η=0.1,α=0.8 and γ=0.8.

Following the parameters in figure 2, we present the frequencies of the three strategies to investigate how they influence each other. In pure Q-learning agents populations (θ=0), punishers are nearly extinct at the early stage, while cooperators and defectors roughly split the population evenly. As time evolves, defectors begin to dominate the population owing to a pay-off advantage. Some cooperators transform into punishers, which effectively suppresses the further spread of defection, although defectors still outnumber cooperators in the steady state (figure 3a). When the density of conformists in the population is 40% (θ=0.4), at the corresponding stage, punishers completely suppress the expansion of defectors, leading to a gradual decline in defectors and a steady increase in cooperators (figure 3b). In addition, the increase in punishers among Q-learning agents leads more conformity agents to adopt cooperation, resulting in a noticeable decline in defectors within the two populations (figure 3e,g). When the density of conformists reaches 80% (θ=0.8), the frequency of punishers decreases to a certain level. In addition, cooperators and defectors reach a near balance, with defectors maintaining a slight advantage (figure 3c). This slight advantage arises because Q-learning agents who seek to maximize pay-offs based on experience tend to defect. Consequently, a small proportion of Q-learning agents guide conformity agents to follow and evolve into a population where defectors prevail (figure 3f, h). Through comparison, we find that changes in the frequency of Q-learning agents always precede those of conformists, with Q-learning agents acting as leaders, while conformists serve as followers and maintainers.

**Figure 3.**
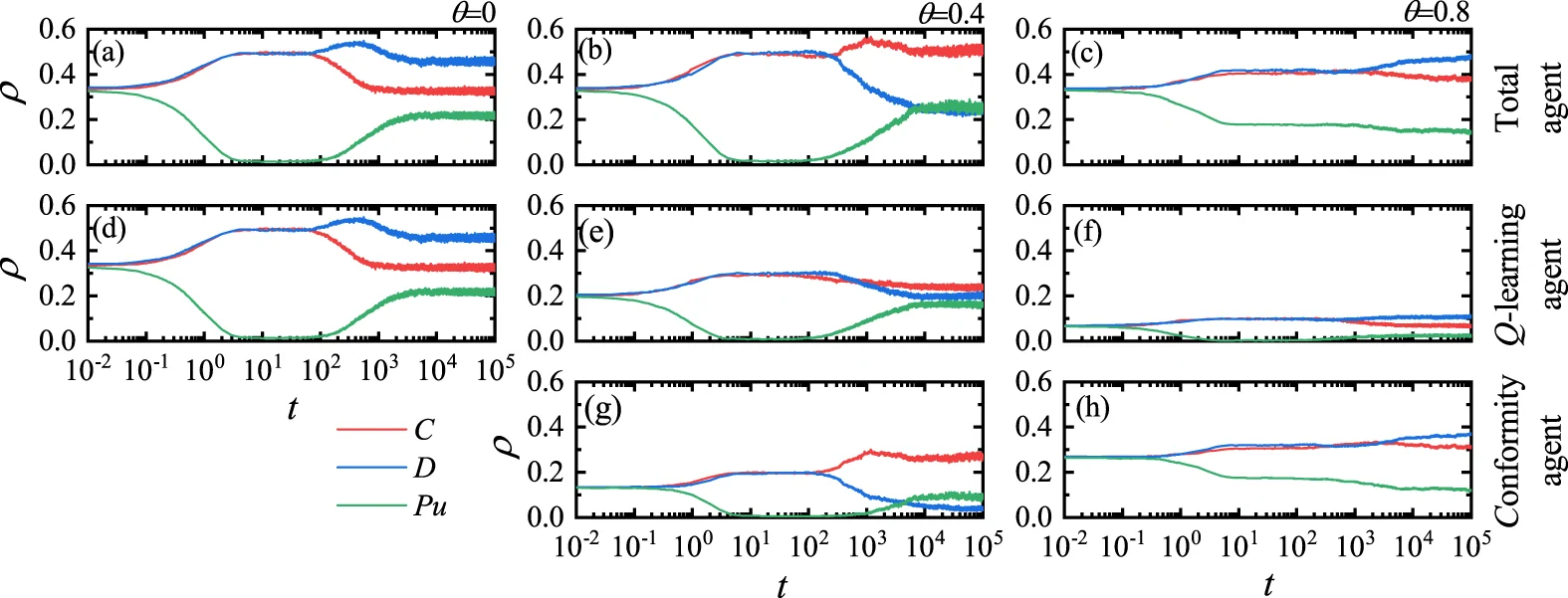
Time dependence of strategies’  frequency reveals conformity agents’  positive and negative incentive effects on the punishment. The top row shows the temporal evolution of the frequencies of three strategies in the whole population under different densities of conformity agents: (a) θ=0, (b) θ=0.4 and (c) θ=0.8. The middle row shows the corresponding frequencies among Q-learning agents for (d) θ=0, (e) θ=0.4 and (f) θ=0.8, while the bottom row shows those among conformity agents for (g) θ=0.4 and (h) θ=0.8. All results are obtained for β=0.5,η=0.1,α=0.8 and γ=0.8.

To explain the impact of different densities of conformist agents on cooperative behaviour from a microscopic perspective, figure 4 presents snapshots of evolutionary dynamics, which illustrate the local development of different strategies across spatial and temporal dimensions. As time evolves, the presence of conformist agents gives rise to cluster formation. These clusters typically exhibit a structure, with conformist agents occupying the central positions and Q-learning agents distributed around the clusters. When a small number of conformist agents are present (figure 4, top row), Q-learning agents act as a sort of membrane or insulation that isolates the clusters formed by conformists. Leveraging the cooperative advantage of these conformist-based clusters and the gradual emergence of punishers, Q-learning cooperators gradually expand outward. The synergistic effect of Q-learning and conformity greatly enhances the threatening effect of punishment on defectors. As the number of conformist agents increases and Q-learning agents become fewer (figure 4, bottom row), the isolating effect of Q-learning diminishes. Eventually, clusters emerge with three strategies evenly distributed. Within these structures, clusters of cooperators are surrounded by defectors from the Q-learning population, while Q-learning cooperators are scattered across the clusters and fail to form a dominant cooperative network, leading to their gradual erosion. These results show that lower densities of conformity agents foster the formation of clusters that enhance the effectiveness of punishment against defectors and support cooperator invasion, but when the density of conformity agents is high, the punishers form clusters that cannot effectively punish defectors. To understand the reasons why the cooperation level varies with θ from a micro perspective, we analyse the probability of the strategy transition under different θ and b in figure 5. The CC links become more prevalent as the proportion of conformists increases, reaching a peak at around θ= 0.4–0.5, after which they gradually decline. Conversely, DD links exhibit a reverse pattern, and when the population is fully composed of conformists, the frequencies of both types of links converge to approximately 30%. The PP links constitute a small fraction of the total connections in the population, but their frequency increases with the growth of θ. Heterogeneous links (CD, CP and DP) gradually decrease as the number of conformists increases, which can be attributed to the tendency of conformists to form clusters. The presence of punishers prevents defectors from gaining an advantage through defection, thereby forcing defectors to change their strategies to obtain higher pay-offs. When θ=0.8, although the number of punishers increases, they tend to form clusters and thus fail to punish defectors directly. As a result, the number of defectors increases again, leading to the lowest overall cooperation level at this stage. When conformists constitute the majority of the population, the frequency of paired connections exhibits oscillations owing to the lack of behavioural preference among conformists, resulting in instability in the population's composition. The frequency of paired connections and the transition probability of strategies are robust against variations of b from the comparison of figure 5A–C.

**Figure 4.**
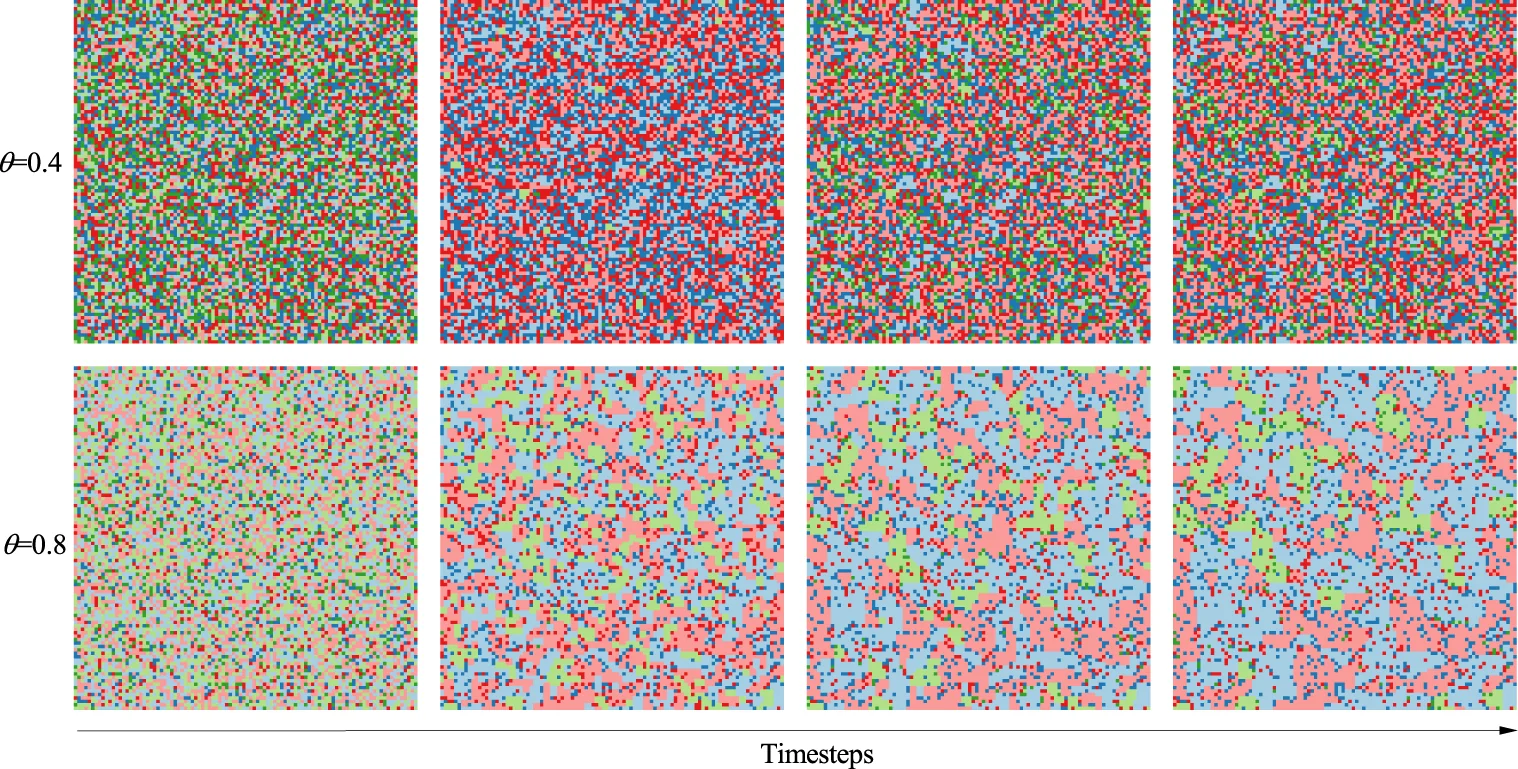
Snapshots of the evolutionary dynamics provide a detailed view of the interactions among different agent types. In the figure, light-coloured nodes represent conformist individuals, whereas dark-coloured nodes represent Q-learning individuals. For both types, red indicates cooperators, blue indicates defectors and green indicates punishers. When θ=0.4, punishers’ clusters and cooperators’ clusters gradually increase in size, and defectors’ clusters gradually decrease (top row). In these clusters, conformity agents occupy the central positions, while Q-learning agents are located at the periphery. When θ=0.8, the conformists gradually form large clusters with a few outlier Q-learning agents scattered within the clusters (bottom row). All results are obtained for β=0.5,η=0.1,α=0.8 andγ=0.8.

**Figure 5.**
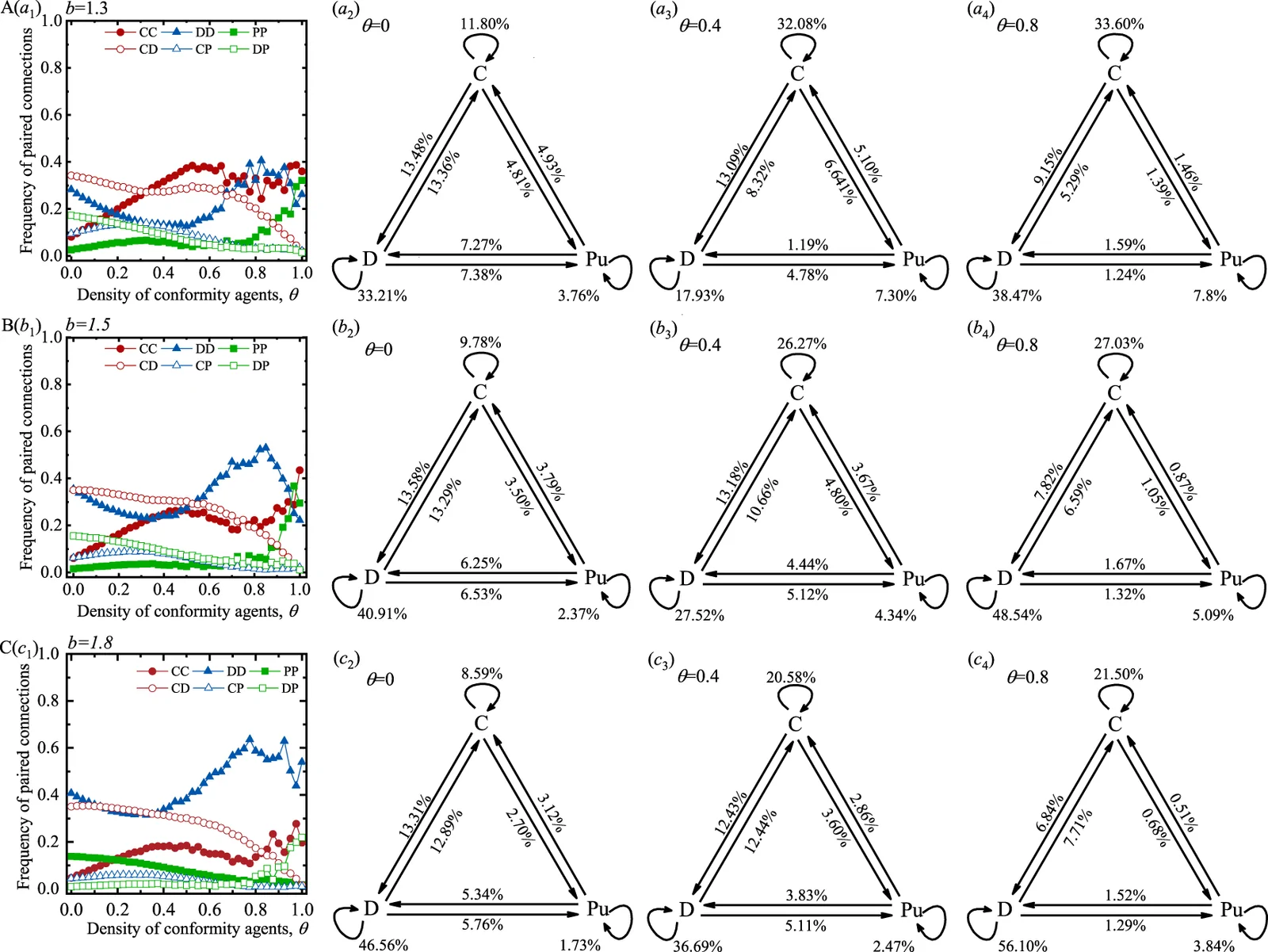
A moderate density of conformists facilitates the persistence of cooperators while weakening the maintenance of defectors. By contrast, when conformists constitute the majority, these three types of strategies tend to maintain their original strategies, resulting in a decline in interaction between heterogeneous strategies. Panels (A)–(C) illustrate how the frequency of pairwise connections and the transition probability of strategies vary with the density of conformist agents θ under b=1.3, b=1.5 and b=1.8. All results are obtained for β=0.5,η=0.1,α=0.8 and γ=0.8.

To further understand the origin of the optimal mixing ratio, we investigated the strategy transition probabilities of the two agent types as a function of θ at b=1.3, as shown in figure 6. In the top row, the probabilities of C→Pu and Pu→Pu increase with θ and reach their maxima at around θ≈ 0.4, while the probability of D→D attains its minimum at approximately the same point. In addition, the probability of C→C continues to increase and reaches a maximum at around θ≈ 0.6. However, when θ>0.4, this advantage is offset by the simultaneous increase in D→D. As a result, the overall cooperation level is maximized near θ≈ 0.4. Examining the two subpopulations separately reveals distinct roles of the updating rules. For θ< 0.5, Q-learning agents promote the persistence of cooperation, as reflected by the increasing probability of C→C, which in turn drives a similar increase among conformity-driven individuals. During this process, the probability of D→D gradually decreases, suppressing the expansion of defection. By contrast, when θ > 0.5, the influence of Q-learning agents progressively weakens, and the overall evolutionary dynamics becomes dominated by conformity-driven updating. Under this regime, individuals tend to maintain their current strategies, leading to fewer transitions between different states. These results suggest that the optimal cooperation level emerges from a balance between the exploratory adaptation of Q-learning agents and the stabilizing effect of conformity-driven individuals.

**Figure 6.**
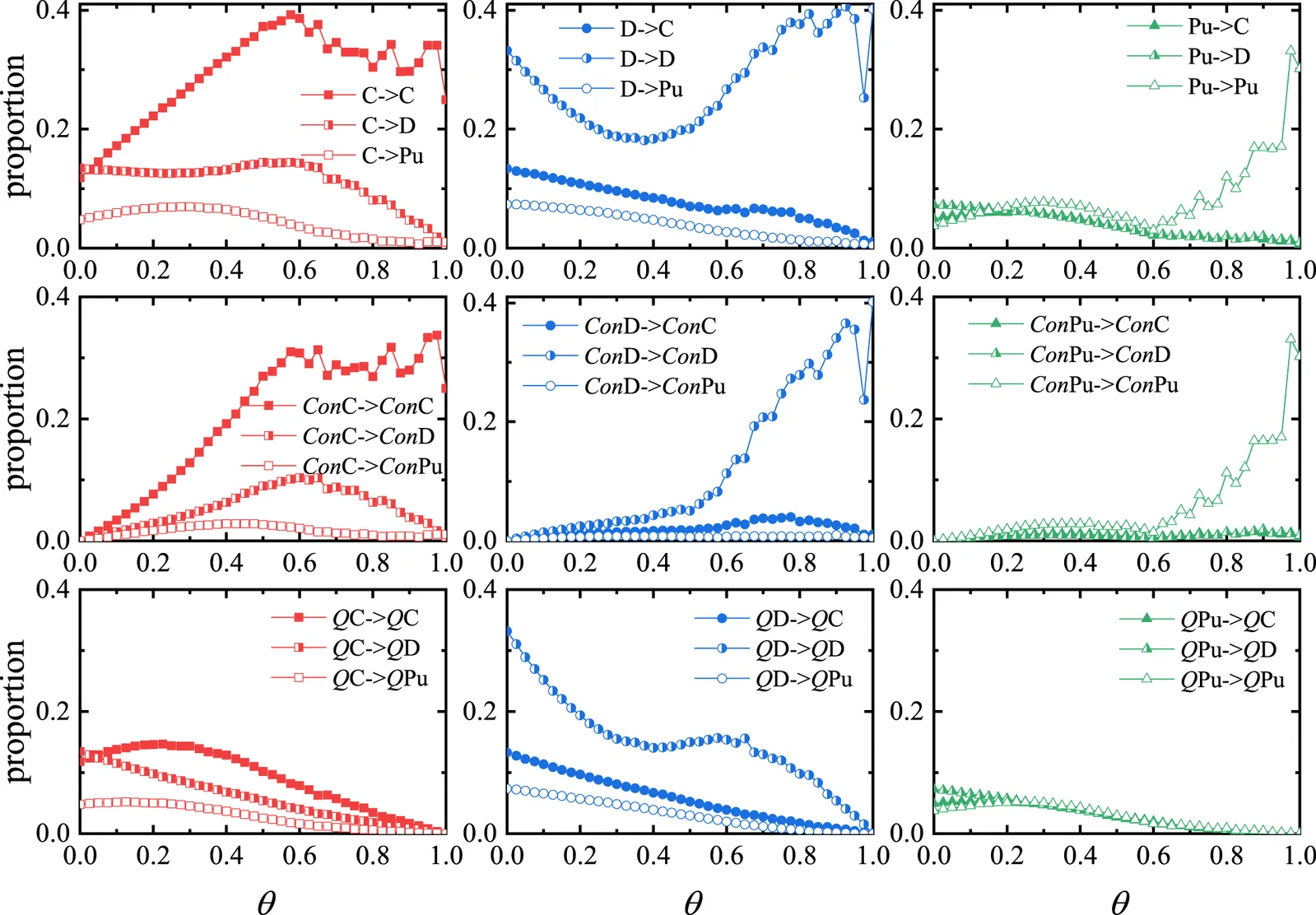
Strategy transition probabilities as a function of θ at b=1.3. The first row shows the transition probabilities for the entire population, while the second and third rows correspond to the conformity-driven and Q-learning subpopulations, respectively. All results are obtained for β=0.5,η=0.1,α=0.8 and γ=0.8.

To further validate the influence of conformist density on agents’ behaviour, we compared the number and size of clusters formed under varying densities of conformist agents in figure 7. It can be observed that the number of clusters of the three strategies is large when θ is small, and the size of clusters is relatively small, indicating that agents adopting the same strategy fail to generate collective strength and remain spatially dispersed figure 7a,b. As θ increases, the sizes of cooperative clusters gradually increase, while their number decreases. This implies that large cooperative clusters emerge, enabling cooperators to resist the invasion of defectors. However, as the densities of conformists increase, the size of cooperative clusters decreases, while defector clusters expand. Eventually, when all agents are conformists, the number and size of clusters for all three strategies become almost equal, reflecting the neutral nature of conformists. To further characterize the roles of the two agent types in cluster formation, we separately analysed clusters centred on conformity-driven individuals and Q-learning agents. As shown in figure 7c,d, the number of conformity-centred clusters is smaller than that of Q-learning-centred clusters. However, their average size is approximately three orders of magnitude larger. This indicates that conformity-driven individuals tend to aggregate into a few large and compact clusters, whereas Q-learning agents are distributed across numerous small clusters. Note that the average size of conformity-centred clusters exceeds the average cluster size of the entire population. Since Q-learning agents tend to form numerous small clusters, their inclusion lowers the whole population average cluster size. These results suggest that incorporating a moderate density of conformists can help cooperators form clusters to maintain cooperation. However, the presence of Q-learning agents is essential to introduce behavioural bias to guide the population; otherwise, it would degenerate into an unorganized crowd.

**Figure 7.**
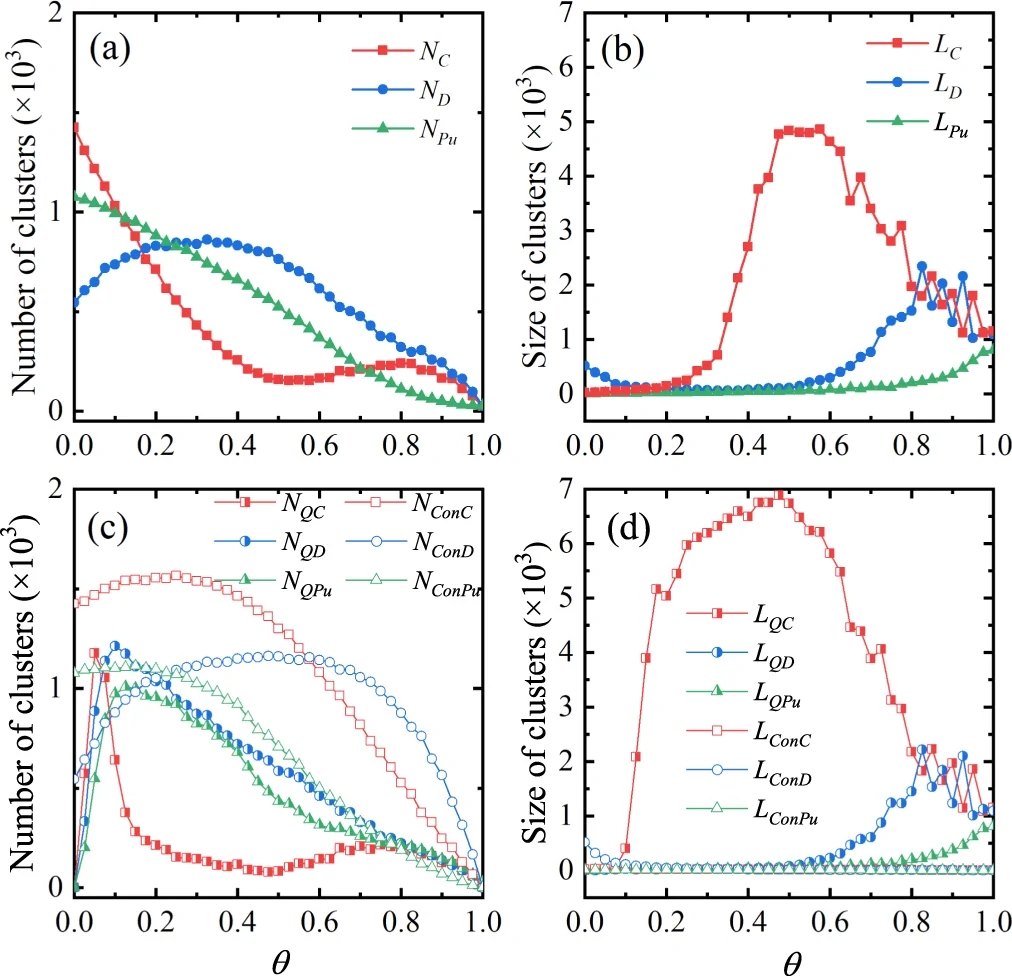
Effects of conformity density on cluster formation. Panels (a) and (b) show the total number and average size of clusters in the population, respectively. Panel (c) presents the number of clusters centred on Q-learning agents and conformity-driven agents. Panel (d) presents the size of clusters centred on Q-learning agents and conformity-driven agents, respectively. The results indicate that conformity-driven individuals contribute most significantly to cluster formation and growth. All results are obtained for β=0.5,η=0.1,α=0.8 and γ=0.8.

Furthermore, we explore how the punishment parameters influence the fraction of cooperation and punishment. As shown in figure 8, larger punishment fines generally lead to higher cooperation levels, whereas increasing punishment costs reduces cooperation. Moreover, the sensitivity of cooperation to punishment parameters is considerably stronger at θ=0.4 than at θ=0.8, indicating that punishment is most effective to restrain defectors near the optimal mixing ratio. At θ=0.8, cooperation remains affected by punishment parameters. However, the correlations become considerably weaker and more irregular than those observed at θ=0.4. Although punishment parameters continue to influence cooperation, their impact becomes weaker and less systematic, exhibiting noticeable fluctuations. This suggests that conformity-driven updating shapes collective behaviour. The results suggest that punishment achieves its maximal effectiveness for a moderate density of conformity agents, while an excessive number of conformity agents diminishes the influence of punishment on evolutionary dynamics.

**Figure 8.**
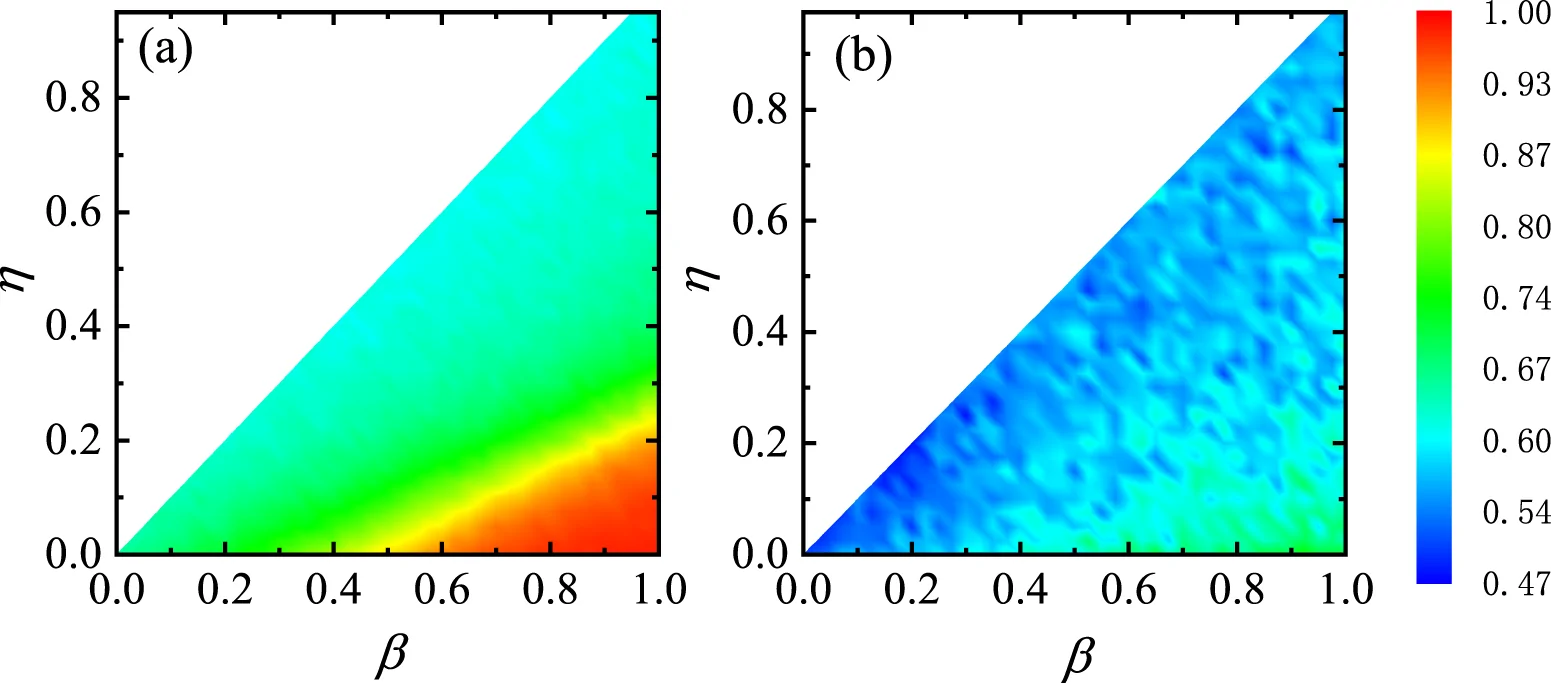
Fraction of cooperators and punishers as a function of the punishment fine β and punishment cost η for (a) θ=0.4 and (b) θ=0.8. Increasing the punishment fine generally promotes cooperation, whereas increasing the punishment cost suppresses it. This effect is more pronounced at θ=0.4, while the influence of punishment parameters becomes weaker at θ=0.8. All results are obtained for b=1.3,α=0.8 and γ=0.8.

Finally, to examine whether our findings are universal, we conducted simulations on two different network structures (a regular small-world network and a scale-free network) and obtained the b–θ phase diagrams in figure 9. The results are highly consistent with contour plots shown in figure 1a, particularly for the regular small-world network. This is because the rewiring of short edges in the regular small-world network does not change the node degree but only alters the network diameter. By contrast, the influence of θ is less pronounced on the scale-free network. This is because the scale-free topology completely changes the node degree distribution—approximately 20% of nodes have very high degrees—which significantly facilitates the clustering of conformist agents. As a result, the overall population becomes highly unstable. Especially, when the majority of agents are conformists, the system is less affected by pay-off differences and more dependent on the initial strategy distribution. Consequently, the final state of the population becomes essentially random, leading to the irregular distribution observed on the right-hand side of figure 9b. Importantly, the observed inhomogeneity in figure 9b is not caused by a lack of convergence of the system. Rather, it is an inherent consequence of the stochastic nature of conformity-driven updating rules [61,62]. The heterogeneous network topology amplifies these local fluctuations, leading to persistent non-uniform patterns in the stationary state.

**Figure 9.**
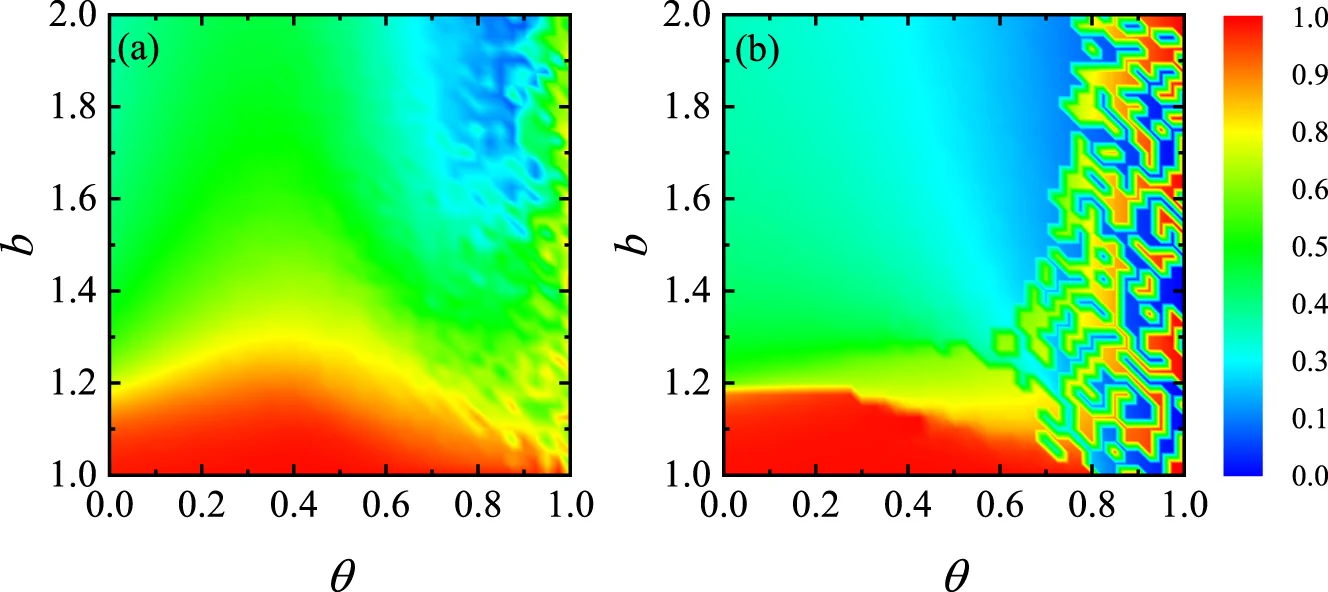
Comparison of the proportions of cooperators and punishers varying with b and θ for (a) regular small-world network and (b) scale-free network. The parameters of networks are $N=10\;000\text{ and }\bar{k}=4$. All results are obtained for β=0.5,η=0.1,α=0.8 and γ=0.8.

## 4. Conclusions

In this study, we investigate the evolution of cooperation in weak prisoner's dilemma games with punishment under two distinct update rules: Q-learning and conformity-driven update rules. We find that the coexistence of two update rules in an appropriate proportion maximizes the effectiveness of punishment in promoting cooperation, compared with the scenario of pure single update rules. First, without conformists, punishment can restrict defectors’ expansion within a manageable range, but its effectiveness remains limited. Second, the introduction of a moderate density of conformity-driven agents significantly improves the cooperation level. The underlying reason is the synergistic effect generated by their complementary roles: conformists forming protective clusters and Q-learning agents driving punishment-mediated expansion when θ=0.4. Taken together, the interactions between these two types of agents allow cooperation to become the dominant strategy with the help of punishment. However, when conformity agents become overly abundant θ=0.8, punishers tend to form isolated clusters and are no longer able to interact broadly with defectors, thereby losing their effectiveness in imposing punishment. Furthermore, to verify the generality of our findings, we further evaluated the performance of our model under various punishment parameters and different network structures.

Our study reveals the complementary roles of two types of agents with distinct characteristics in promoting cooperation, offering new insights to understand the emergence of cooperation. Moreover, our findings extend beyond human–human interactions and provide a theoretical basis for understanding cooperation in human–machine systems. Specifically, the Q-learning update rule models machines equipped with independent cognitive abilities but lacking prosocial traits, whereas the conformity update rule captures the prosocial behaviour of humans as inherently social beings. From this perspective, machines exhibit a stronger inclination towards exploration and adaptation, whereas conformist individuals are more likely to form clusters. Furthermore, when the proportion is appropriate, the cooperative behaviour in human–machine hybrid settings can be enhanced. Future research can explore human–machine interaction from multiple perspectives, including richer cognitive heterogeneity between humans and agents.

## Data Availability

The code used in the study is freely available at [63].
